# The feasibility and acceptability of a peer-support group for mental health in Filipino prisons

**DOI:** 10.1192/bjo.2021.747

**Published:** 2021-06-18

**Authors:** Bethany Platford

**Affiliations:** Nuffield Centre, Faculty of International Health and Development, School of Medicine, University of Leeds

## Abstract

**Aims:**

To assess the feasibility and acceptability of implementing and facilitating a peer-support group for mental health in Filipino prisons.

**Objectives:**

To identify the logistical issues faced in implementing and facilitating healthcare in Filipino Prisons

To explore attitudes of potential participants towards the implementation of a peer-support group for mental health in the prisons

To provide logical recommendations from my findings to inform future mental health support for prisoners in the Philippines

**Method:**

Ethical approval was granted by the University of Leeds prior to data collection. In-country ethical approval was granted through my host Dr Rachael Pickering. Data collection occurred through observations and semi-structured interviews. Participants recruited included six secure-environment healthcare workers, eight prisoners and six ex-prisoners. Both prisoners and ex-prisoners were identified through gatekeepers and informed consent was gained. Interviews were transcribed before coding and themes identified.

**Result:**

Feasibility: Bureaucracy and corruption were the main barriers to the potential successful implementation of a peer-support group, which were emergent themes. Space, time and staff were all themes identified that may help facilitate the group.

Attitudes: It was noted that there is a significant lack of knowledge surrounding what mental health is and its causes. Stigma and discriminatory actions were also noted by many participants as barrier to the group. However, seven prisoners and five ex-prisoners said they would join.

**Conclusion:**

The findings highlighted many barriers but with perservance and local cultural competence this peer-support group could be feasible and be accepted in Filipino prisons. It will be reducing a high unmet need for mental health services in these prisons and if ran successfully with positive effects, will be an example for other prisons across the Philippines and other low-middle income countries.

